# Acute Kidney Injury After Open Heart Surgery

**DOI:** 10.7759/cureus.25899

**Published:** 2022-06-13

**Authors:** Iskander S Al-Githmi, Abdullah A Abdulqader, Abdulrahman Alotaibi, Badr A Aldughather, Omar A Alsulami, Sahal M Wali, Muath S Alghamdi, Tarig S Althabaiti, Talal B Melebary

**Affiliations:** 1 Thoracic Surgery, King Abdulaziz University Hospital, Jeddah, SAU; 2 General Practice, King Abdulaziz University Hospital, Jeddah, SAU; 3 General Surgery, King Abdulaziz University Hospital, Jeddah, SAU; 4 Neurosugery, King Abdulaziz University Hospital, Jeddah, SAU; 5 Pediatrics, King Abdulaziz University Hospital, Jeddah, SAU

**Keywords:** coronary artery bypass graft surgery, risk injury failure loss end-stage (rifle), kau hospital pahala, kauh, kidney disease improving global outcomes (kdigo) classification, esrd, cardiac surgery, cabg, akf, aki

## Abstract

Introduction:* *Acute kidney injury (AKI) is a term used to describe when the kidney loses its function rapidly. And it’s associated with an increase in the level of serum creatinine by 0.5 to 1mg/dL. It can be diagnosed by a plethora of criteria such as the Kidney Disease Improving Global Outcomes (KDIGO) and the Risk, Injury, Failure, Loss, End-stage (RIFLE) criteria. Cardiac surgery-associated AKI (CSA-AKI) is the most prevalent complication in patients following cardiac surgery and is also linked to increased mortality and morbidity rates. In addition, exogenous and endogenous toxins, ischemia and reperfusion, inflammation, oxidative stress, metabolic factors, and neurohormonal activation may all play a role in the development of CSA-AKI. All these factors may be active at varying time intervals and with different degrees of intensity, or may function simultaneously.

Methods: In late 2019, a retrospective study was conducted by reviewing the health data of patients who underwent coronary artery bypass graft (CABG), valvular repairs, and other open cardiac surgeries at the King Abdulaziz University Hospital (KAUH), Jeddah, Saudi Arabia, between November 2014 and June 2019. Information was obtained from the Hospital information system, Jeddah, Saudi Arabia. Of the 159 patients who underwent open-heart surgery at KAUH, 126 (79.2%) were male and 33 (20.8%) were female. Patients below 15 years of age and those with poor renal function prior to open cardiac surgery were excluded. The KDIGO criteria were used to diagnose AKI for our patients.

Results: In this study, 34% of the patients experienced AKI after open cardiac surgery, and the most frequent risk factor encountered was diabetes mellitus (DM), which was present in 97 (61%) patients, followed by angina pectoris in 93 (58.5%) patients. Hypertension was identified in 85 (53.5%) and acute myocardial infarction in 82 (51.6%) patients. There were only two (1.3%) patients with known cases of chronic obstructive lung disease (COPD). Of the surgeries, 131 (82.4%) were classified as elective and 28 (17.6%) were urgent.

Conclusion: The most common risk factor associated with AKI following open-heart surgery is DM, followed by angina pectoris. However, further studies are required to investigate all the cardiac procedures.

## Introduction

Acute kidney injury (AKI) is characterized by a rapid loss of renal function and is associated with a continuous increase in serum creatinine levels by 0.5-1 mg/dL (a related elevation of 50% or continuous elevation of 0.5 to 1 mg/dL), according to a study by Makris et al. [1]. According to Kellum et al., the disease can be diagnosed using a plethora of criteria after cardiac surgery, such as the Kidney Disease Improving Global Outcomes (KDIGO) and the Risk, Injury, Failure, Loss, End-stage (RIFLE) criteria. According to the KDIGO criteria, AKI is defined by any of the following: an elevation in the serum creatinine level of ≥0.3 mg/dl (≥26.5 µmol/l) within two days, urine volume <0.5 ml/kg/h for six hours or more, or elevation in the serum creatinine level to ≥1.5 × baseline, which occurred during the past seven days or may have been predicted [2]. The RIFLE criteria classify the patients as at risk for kidney failure (glomerular filtration rate (GFR) decrease of >25%, elevation in serum creatinine by 1.5×, or urine output of <0.5 mL/kg/h for six hours), kidney injury (GFR decrease of >50%, elevation in serum creatinine by 2×, urine output of <0.5 mL/kg/h for 12 hours), renal failure (GFR decrease of >75%, three-fold increase in serum creatinine level, or serum creatinine >4 mg/dL (350 µmol/L) in cases of severe increase of at least 0.5 mg/dL (44 µmol/L), urine output <0.3 ml/kg/h for 24 h (oliguria) or anuria for 12 hours), renal function loss (kidney function is completely lost for >4 weeks), and end-stage renal disease (ESRD), in which kidney function is completely lost for >12 weeks [3]. 

Globally, more than two million cardiac surgeries are conducted every year, with the prevalence of cardiac surgery-associated AKI (CSA-AKI) ranging from 5% to 42%. According to a statistical analysis, CSA-AKI is the most prevalent complication in patients following cardiac surgery and is also linked to increased mortality and morbidity rates [3]. In addition, exogenous and endogenous toxins, ischemia and reperfusion, inflammation, oxidative stress, metabolic factors, and neurohormonal activation may all play a role in the development of CSA-AKI. All these factors may be active at varying time intervals and with different degrees of intensity, or may function simultaneously [4].

According to a study by Bellomo et al. (2006), up to one-third of heart surgery patients had AKI [5], and a majority of patients who developed AKI were elderly, had more comorbid diseases, had worse preoperative kidney function, and were more susceptible to urgent procedures [6]. Another prospective study of 4374 individuals indicated that even a minor increase in the blood creatinine level (followed by a sharp drop) is related to an increased risk of death [7].

To our knowledge, there is a relationship between the occurrence of AKI after cardiac surgery and the likelihood of encountering ESRD in the Saudi population. Thus, this study aimed to determine the prevalence of CSA-AKI after heart surgery in the Saudi population at King Abdulaziz University Hospital (KAUH), Jeddah, Saudi Arabia, between 2014 and 2019.

## Materials and methods

In late 2019, a retrospective study was conducted by reviewing the health data of patients who underwent open-heart surgery at the King Abdulaziz University Hospital (KAUH), Jeddah, Saudi Arabia, between November 2014 and June 2019. Information was obtained from the system datasheet of the Hospital information system, Jeddah, Saudi Arabia. Of the 159 patients who underwent open-heart surgery at KAUH, 126 (79.2%) were male and 33 (20.8%) were female.

All post-operative patients were transferred to the ICU for intensive monitoring; and were kept under observation, with frequent labs and investigations such as cardiac enzymes, venous blood gas (VBG), arterial blood gas (ABG), chest x-rays, and ECG.

Following the administration of prophylactic antibiotics preoperatively, they were maintained on one gram of cefazolin intravenously every eight hours until the chest tubes were removed. Patients with a mean arterial pressure (MAP) less than 60 mmHg were given inotropes. Dopamine was administered to all individuals whose urine output was less than 30cc/h. Other medications were given including aspirin, statins, and a beta-blocker such as metoprolol.

No nephrotoxic drugs were administered during their hospital stay; however, for analgesia, ibuprofen was given for a maximum of seven days, with the addition of tramadol in some patients.

The inclusion criteria were as follows: ages between 15 and 90 years, with a mean age of 55.22+13.2 years. Patients below 15 years of age and those with poor renal function prior to surgery were excluded. The KDIGO criteria was used to diagnose the included patients [2]. The King Abdulaziz University Institutional Review Board (IRB) granted ethical approval for this investigation with an approval number of 142-21.

Data were analyzed using the IBM SPSS Statistics for Windows, Version 21.0 (Released 2012; IBM Corp., Armonk, New York, United States). Continuous variables were described as mean and standard deviation. To define the categorical variables, we calculated them. The difference between qualitative and quantitative variables was assessed using an independent t-test. To determine the associations between two qualitative and quantitative variables, a chi-square test and correlation analysis were performed, respectively. Statistical significance was set at p<0.05.

This study mainly aimed to determine the prevalence and incidence of acute renal injury following open cardiac surgery at KAUH, Jeddah, Saudi Arabia. Moreover, we aimed to identify pre-, peri-, and postoperative risk factors.

## Results

In this study, 34% of the patients experienced AKI after open cardiac surgery, and the most frequent risk factor was diabetes mellitus (DM), which was present in 97 (61%) patients, followed by angina pectoris in 93 (58.5%) patients. Hypertension was identified in 85 (53.5%) and acute myocardial infarction in 82 (51.6%) patients. There were only two (1.3%) patients with known cases of chronic obstructive lung disease (COPD). Of the surgeries, 131 (82.4%) were classified as elective and 28 (17.6%) were urgent.

The blood pressure (BP) of the patients was taken preoperatively. The mean systolic and diastolic BP were 124.17+23.92 mmHg (minimum, 80 mmHg; maximum, 200 mmHg) and 67.3+16.14 mmHg (minimum, 29 mmHg; maximum, 120 mmHg), respectively. The mean random blood sugar level was 8.78+3.97 mmol/L, (minimum, 4.0 mmol/L; maximum: 21.4 mmol/L). The mean ejection fraction was 49.92+9.82%, (minimum, 25%; maximum: 73%). Data on 47 patients were missing. The mean preoperative laboratory results of the patients were as follows: serum creatinine (CK), 91.12+33.15 (minimum, 28.1; maximum, 287); blood urea nitrogen (BUN), 5.48+2.5 (minimum, 1.7; maximum, 17); hemoglobin (Hb), 12.81+2.18 (minimum, 8; maximum, 17.8).

The mean postoperative CK was 106.17+36.5 (minimum, 29; maximum, 267), and that of males and females were 110+36.5 and 91.3+32.9, respectively. There was no significant difference in the age group and BMI between groups. Moreover, there was no relation between postoperative CK, DM, and atrial fibrillation. The mean postoperative BUN was 5.64+2.4 (minimum, 1.8; maximum, 16.4), and the mean postoperative Hb was 9.8+1.54 (minimum, 6; maximum, 16). Postoperative Hb had a significant relationship with postoperative CK (P=0.032), and there was also a positive relationship between the number of RBC transfusion units (RBCU) and postoperative CK (P=0.26). The correlation between the age and RBCU was also statistically significant (P=0.028).

Despite the number of missing intraoperative variables (104/159 patients) for both cross-clamping time and bypass time, the mean for the former was 58.57+19.5 min (minimum, 19 min; maximum, 124 min). The mean for the latter was 96.05+31.56 min (minimum, 28 min; maximum, 240 min). A total of 102 (63.7%) patients received a blood transfusion, with a maximum of six units in two (2.9 %) patients. Referring to mortality measurements, the average duration of ICU stay was 73.7+73.5 hours (minimum, 3 hours; maximum, 648 hours). The mean duration of hospital stay was 12.1+14.35 days (minimum, one day; maximum, 174 days). Only two patients had atrial fibrillation. Regarding medication, 129 (81.1 %) patients were on beta-blockers, 100 (62.9%) were on angiotensin-converting enzyme (ACE) inhibitors, 36 (22.6%) were on calcium channel blockers, 141 (88.7%) were taking diuretics, and 113 (71.1%) were on statins.

**Table 1:** Pre- and postoperative serum creatinine and BUN levels BUN: blood urea nitrogen; HTN: hypertension; DM: diabetes mellitus; AMI: acute myocardial infarction; COPD: chronic obstructive pulmonary disease

		Preoperative serum creatinine	Postoperative serum creatinine	Preoperative blood urea nitrogen	Postoperative blood urea nitrogen
Sex	Male	94.80	110.07	5.56	5.73
	Female	77.08	91.32	5.22	5.35
Classification of surgery	Elective	90.80	105.68	5.39	5.55
	Urgent	92.64	108.51	5.96	6.10
HTN	Yes	97.98	112.09	6.11	6.28
	No	83.24	99.39	4.78	4.92
DM	Yes	91.79	108.79	5.70	5.91
	No	90.08	102.10	5.16	5.24
AMI	Yes	94.08	109.99	5.83	6.05
	No	87.21	101.56	5.07	5.19
Angina pectoris	Yes	90.48	106.82	5.32	5.54
	No	92.03	105.27	5.73	5.79
COPD	Yes	80.35	99.20	4.85	4.85

There was a significant relationship between the time of bypass and the postoperative hemoglobin (Hb) level (P=0.001). There was no relationship between the time of bypass and patient age (P=0.35), sex (P=0.757), BMI, blood transfusion (P=0.893), and presence of DM (P=0.438) or hypertension (P=0.159). There was a significant relationship between increased preoperative BUN level and ICU stay (P=0.003). The duration of hospital stay was significantly related to preoperative Hb level, preoperative BUN level, and bypass time (P=0.043, P=0.002, and P=0.001, respectively). There was also a significant relationship between mortality, according to the days in the ICU and preoperative BUN (P=0.019). In contrast, there were no significant relationships between mortality and age (P=0.938), preoperative serum creatinine (P=0.657), preoperative ejection fraction (P=0.112), bypass time (P=0.353), and cross-clamping time (P=0.101). There was also no significant relationship between the cross-clamping time and postoperative serum creatinine (P=0.138) or BUN (P=0.483).

## Discussion

In this study, there was a significant relationship between the bypass time and the postoperative Hb level (P=0.001). However, patients with cardiopulmonary bypass (CPB) times longer than 90 minutes, as measured by sensitive kidney-specific proteins, showed more significant kidney damage than did patients with CPB times shorter than 70 minutes, according to a prospective research study conducted in Germany [8].

In the study by Wittlinger, aging was identified as a separate risk factor [9], which was similarly found in our study.

Notably, age remains to be a debatable risk factor; while some studies and this study found that the chances of developing acute renal failure in older patients are high, other studies disagreed [10-13]. Because of the functional reserve loss in aging kidneys, older people are prone to several types of acute renal failure. In addition, reduced circulation may make it difficult for elderly patients to cope.

In this study, 34% of the patients experienced acute renal injury after open cardiac surgery. However, a previous study found that the prevalence of AKI in male patients was higher than that in female patients [14].

In 2019, a retrospective study conducted on 115 patients found a significant relationship between postoperative AKI and time of bypass, as an increase in bypass time by 10 seconds increases the risk of postoperative AKI by 17.1% [15]. The findings of the present study concur with that of the previously mentioned retrospective study. However, other studies disagree with this result and find no relationship between the time of bypass and risk for postoperative AKI. For instance, Mancini et al. found no relationship as they stratified participants according to the time of bypass [16]. An association between perioperative anemia during heart surgery and AKI has been identified in several studies [17]. However, the mean preoperative Hb was 12.81.

In a study published by Habib et al., many factors play a role in the development of AKI post-CPB in anemic patients; the most popular theories revolve around inflammatory processes, embolic phenomena, and ischemia-induced endotoxin release; however, because this field is still poorly understood, more research is needed [18]. 

In this study, 62.9% (100) of patients were treated with ACE inhibitors. A study has shown that the long-term use of ACE inhibitors is significantly associated with post-heart surgery AKI [19], which could explain the occurrence of AKI in our study.

Additionally, in this study analysis, the duration of hospital stay was significantly related to preoperative Hb level, preoperative BUN level, and bypass time (P=0.043, P=0.002, and P=0.001, respectively). This may have played a role in increasing the risk of AKI after cardiac surgery; consequently, the overall hospital stay was prolonged. Furthermore, there are some studies that agree by showing that "the stay in the ICU for patients with postoperative AKI was fourfold than that in patients without AKI,” indicating increased hospital resources utilization [13].

Despite improvements in medical technology, cardiac surgery continues to be a high-risk procedure with a plethora of postoperative complications, such as AKI [20]. Based on previous research, the risk factors linked to AKI following open cardiac surgery were preoperative age and chronic illnesses, such as DM, HTN, angina pectoris, myocardial infarction, and COPD.

In a study by Patschan and Müller, DM is speculated to cause AKI through a variety of pathways that are influenced by factors such as an increase in reactive oxygen species (ROS), inactivation of nitric oxide (NO), and the production of free fatty acids due to a hyperglycemic milieu [21].

Szczech et al. reported that in cases of acute hypertension with a low estimated glomerular filtration rate (eGFR) at baseline, there is a hindrance in compensatory filtration which further reduces eGFR, raises creatinine levels, and eventually leads to the development of AKI [22]. 

According to Wu et al., the pathogenesis of COPD-associated AKI is unknown and requires additional research. To improve the quality of treatment and the well-being of these patients, they advocate effective respiratory disease control, early detection of cardiac disease, and limiting inflammatory processes [23]. Furthermore, inflammation also plays a role in acute coronary syndrome (ACS)-induced AKI via numerous mediators such as IL-6, ET-1, and NT-ProBNP, in a paper by Ortega-Hernández et al. [24].

Abnormal preoperative measurements included BP, serum creatinine (mL/min), Hb (g/dL), ejection fraction (%), and BUN. Perioperative risk factors include CPB time and duration of cross-clamping [9,25-27]. Statistically, we analyzed these variables and found significant relationships with postoperative AKI cases.

Patients with impaired kidney function before surgery needed perioperative courses that were more difficult and linked to a higher in-hospital death rate. This was confirmed by many earlier investigations, which showed that kidney function impairment before surgery is a risk factor for postoperative AKI following cardiac surgery [10,13,28]. In contrast, this link between kidney function before surgery and postoperative impairment in kidney function was not observed in a study of 843 individuals [29]. This difference could be explained by several factors, including perioperative patient care, patient selection, and the criteria used for postoperative kidney function degradation.

This study is unique, as it is the first such study conducted in KAUH. Nevertheless, there are some limitations. One limitation is that there were insufficient studies on postoperative AKI following open cardiac surgery in our nation. The other limitations are that this retrospective study was conducted in a single center, and as such had a limited number of participants and some missing data. Therefore, to overcome these limitations, studies with increased sample sizes are recommended.

## Conclusions

In conclusion, AKI is considered one of the most dangerous complications affecting more than a third of our included patients postoperatively, impacting both morbidity and mortality. DM, HTN, Angina pectoris, and prolonged CPB times are risk factors associated with postoperative AKI. Moreover, this urges the need for frequent monitoring of blood glucose levels preoperatively, controlling blood pressure, maintaining CPB time below 70 minutes, and assessing cardiac risk factors. These are key in preventing postoperative AKI. Furthermore, additional studies are required to investigate all the cardiac procedures and address the multiple perioperative risk factors.
